# Traditional Chinese Medicine Shi-Bi-Man regulates lactic acid metabolism and drives hair follicle stem cell activation to promote hair regeneration

**DOI:** 10.1186/s13020-023-00791-z

**Published:** 2023-07-15

**Authors:** Haojie Du, Tao Zhang, Qiao Wang, Xinran Cao, Huiwen Zheng, Jiabin Li, Jianxia Zhu, Jiao Qu, Lehang Guo, Yang Sun

**Affiliations:** 1 State Key Laboratory of Pharmaceutical Biotechnology, School of Life Sciences, Nanjing University, 163 Xianlin Avenue, Nanjing, 210023 China; 2 Department of Ultrasound, Shanghai Tenth People’s Hospital, Shanghai, China; 3 Department of Dermatology, Children’s Hospital, Zhejiang University School of Medicine, National Clinical Research Center for Child Health, Hangzhou, 310052 Zhejiang Province China; 4 Shenzhen Sipimo Technology Co., Ltd., Shenzhen, 518000 Guangdong Province China; 5 Jiangsu Key Laboratory of New Drug Research and Clinical Pharmacy, Xuzhou Medical University, 209 Tongshan Road, Xuzhou, 221004 China

**Keywords:** Hair follicle stem cell activation, SBM, Cynomolgus monkey, Single-cell RNA sequencing, LDHA, Laminin pathway

## Abstract

**Background:**

As a supplement for promoting hair health, Shi-Bi-Man (SBM) is a prescription comprising various traditional Chinese medicines. Though SBM has been reported to promote hair regeneration, its molecular mechanism remains unclear. Cynomolgus monkeys (*Macaca fascicularis*) are non-human primates with a gene expression profile similar to that of humans. The purpose of this research is to evaluate the effect of SBM on promoting hair regeneration in cynomolgus monkeys and to reveal the underlying mechanism.

**Methods:**

The effect of SBM on hair regeneration was observed by skin administration on 6 cynomolgus monkeys with artificial back shaving. The molecular mechanism of SBM was studied using single-cell RNA sequencing (scRNA-seq) in combination with quantitative polymerase chain reaction (qPCR) detection for gene transcription level, and immunofluorescence staining verification for protein level.

**Results:**

SBM significantly induced hair regeneration in cynomolgus monkeys, increased hair follicle number and facilitated hair follicle development. ScRNA-seq revealed an increase in the number of hair follicle stem cells (HFSCs) with a higher activation state, as evidenced by the higher expression of activation marker *LDHA* related to metabolism and the proliferation marker *MKI67*. Immunofluorescence analysis at the protein level and qPCR at the mRNA level confirmed the sequencing data. Cellchat analysis revealed an enrichment of ligand-receptor pairs involved in intercellular communication in Laminin-related pathways.

**Conclusion:**

SBM significantly promotes hair regeneration in cynomolgus monkeys. Mechanically, SBM can up-regulate LDHA-mediated lactic acid metabolism and drive HFSC activation, which in turn promotes the proliferation and differentiation of HFSCs.

## Introduction

As the primary shield between the human body and its environment, hair plays a crucial role in the course of human evolution. The protective function of hair extends beyond shielding the skin from harmful UV damage; it also plays a role in aiding the secretion of sebaceous glands and regulating body temperature [1]. Moreover, as an integral component of one's appearance and identity, hair significantly influences an individual's mental wellbeing and social interactions [2].

The mammalian hair-growth cycle consists of three distinct stages—anagen, catagen and telogen—which are regulated by periodic changes of hair follicles [3, 4]. The hair follicle, a complex organ located in the dermis, is a key component of the skin and is composed of inner and outer root sheaths, as well as connective-tissue sheaths, providing nutrients for hair growth. HFSCs are pluripotent stem cells found within hair follicles that possess the potential for self-renewal and multi-differentiation. During the telogen phase, HFSCs remain in a quiescent state. When transitioning to the anagen phase, HFSCs are activated rapidly, leading to proliferation and differentiation, facilitating periodic hair follicle growth and the renewal of the epidermis and sebaceous glands [5]. Therefore, the activation of HFSCs is a crucial step in initiating hair regeneration.

Pathological alopecia is characterized by environmental, emotional, or disease-related factors that cause a shortened anagen phase and prolonged telogen phase in hair growth [6]. At present, the common types of alopecia are seborrheic alopecia and aging induced alopecia. Androgen alopecia (AGA) is a significant contributor to seborrheic alopecia, affecting up to 80% of men by the age of 70. In patients with AGA, HFSCs are unable to receive activation signals and keep quiescent state, leading to hair follicle atrophy and loss of hair regeneration ability. Currently, only two drugs, Finasteride and Minoxidil, are licensed by the United States Food and Drug Administration (FDA) for AGA treatment. However, smear administration for Minoxidil may cause adverse reactions, such as irritant dermatitis, erythema, and itching, while oral administration may affect male sexual function [7]. Consequently, the development of a mild, long-lasting hair growth-enhancing drug would significantly improve patients' life quality and generate substantial economic benefits. Traditional Chinese medicine has various pharmacological effects, such as inhibiting 5α-reductase, improving microcirculation, antagonizing androgens, etc. Its multi-targets and synergistic action of components or prescriptions can effectively improve diseases [8]. Kang et al. reveal Yangxue Shengfa capsule regulates AGA associated genes, including IGF-1, DKK-1, and TGF-β, and promotes cell proliferation of dermal papilla cells [9]. Xiao et al. verify that alpinetin activates HFSCs to promote hair regeneration through Wnt pathway, and has no toxicity to keratinocytes and fibroblasts [10].

Recently, the rapid development of biotechnology, especially omics, has greatly boosted our comprehension of natural product mechanism [11]. SBM is constituted of diverse ingredients derived from natural plants, including ginseng radix, polygonum multiflorum, radix angelicae sinensis, aloe, linseed and green tea extract. Previous research reveals that SBM attenuates D-Gal induced aging and oxidative stress in mice by activating the Nrf2 / Keap1 signaling pathway, suggesting that SBM can act as a promising traditional Chinese medicine to treat diseases induced by aging and oxidative stress. In addition, SBM can promote hair regeneration in mice through the regulation of the FGF signaling pathway in dermal papilla cells in mice, as revealed via single-cell sequencing [12]. Considering the heterogeneity of gene expression levels in mice and humans, we combined single-cell RNA sequencing on cynomolgus monkeys to uncover the underlying mechanism of SBM in promoting hair regeneration. Our findings indicate that SBM regulates LDHA-mediated lactic acid metabolism of hair follicle stem cells, leading to HFSC activation.

## Materials and methods

### Cynomolgus monkeys and reagents

Six cynomolgus monkeys were raised at the monkey breeding base of Changchun Biotechnology Development Co., Ltd., Guangxi, China. Cynomolgus monkeys were anesthetized with 15 mg/kg ketamine intramuscular injection. Electric razor was used for shaving two pieces (5 cm apart) of hair from the back of each monkey, each measuring 2.5 × 2.5 cm. On the left side, 300 μL Shi-Bi-Man (Sipimo Biotechnology Co., Ltd.) were smeared twice daily, in the morning and evening; On the right side, same amount of normal saline was smeared. The treatment was continued for 12 consecutive days, and the photos were taken every morning before the treatment.

On the 12th day, 70 mg/kg pentobarbital sodium solution was injected intravenously for euthanasia. One part of the skin tissue was directly immersed in paraformaldehyde for fixation, followed by paraffin embedding, tissue sectioning, and H&E staining. The other part was used for single-cell RNA sequencing, and the paired samples in the vehicle and SBM group were from the same cynomolgus monkey.

### Single-cell RNA-seq

The skin tissue was placed in a dish containing cold phosphate-buffered saline (PBS), cutting into 0.5 cm × 0.5 cm on the ice and was washed with PBS three times. Then the skin tissue was transferred into a 30 mm plate with 1.5 mL of dispase and kept 4 ℃ overnight. The skin tissue was subsequently cut into small pieces, transferred into a 15 mL centrifuge tube containing 500 μL of 20 mg/mL collagenase 1 together with dispase. The tube was placed horizontally in a 140 rpm 37 ℃ water bath shaker for 30–40 min. The post-digestion cell suspension was passed through a 70 μm sieve (Miltenyi), and 5 mL Dulbecco’s Modified Eagle Medium (DMEM) with 10% FBS was added to wash sieve. The passed cell suspension was centrifuged at 500*g* for 5 min at 4 ℃. Next, 1 mL red cell lysis reagent was added into the cell precipitate and remixed, followed by 2–3 min incubation on ice. 5 mL of DMEM with 10% FBS was added to stop lysis, followed by centrifugation at 500*g* for 5 min at 4 ℃, and 5 mL of DMEM with 10% FBS was added to wash the cell precipitate. The cell pellets were re-suspended in sorting buffer. Single cells captures and scRNA-seq library conduction were performed by Shanghai Xuran Biotechnology and prepared following the manufacturer’s protocol (10 × Genomics).

Single-cell RNA-seq data was analyzed in R (v3.6.0) using the Seurat package (v3.2.3). We filtered cells based on the number of UMIs (less than 500 vs. 6000 UMI) and more than 5% mitochondrial readings. Data was normalized and integrated using the “SCT” normalization method. After performing principal component analysis (PCA), we chose the top 40 principal components (PCs) for uniform manifold approximation and projection (UMAP) dimensionality reduction. Doublets were filtered through the Doublet Finder package (v2.6.0). Seurat’s “FindAllMarkers” function was used to find differentially expressed genes (DEGs) with gene expression considered only for those expressed in at least 25% of the cells.

### Immunofluorescence

Cynomolgus monkeys were sacrificed at day 12. Their back skin was obtained for paraffin sections (6 μm), hematoxylin and eosin (H&E) staining and immunofluorescence analysis.

The sections were blocked with 3% Bovine Serum Albumin (BSA) before incubation with anti-SOX9 (Cell Signaling Technology, 82630T, rabbit, 1:100), anti-LDHA (Cell Signaling Technology, 3582T, rabbit, 1:100) or anti-KI67 (Abcam, ab16667, rabbit, 1:100) antibodies. For immunofluorescence experiments, the corresponding Horseradish peroxidase (HRP)-linked secondary antibodies and fluorophores were used for coating.

### Quantitative reverse transcription PCR

The total RNA of skin tissue was isolated using TRIzol reagent and cDNA was synthesized using iScript Reverse Transcription Supermix (Bio-Rad). ChamQ Universal SYBR qPCR Master Mix (Vazyme) and BioRad CFX96 ouch™ Real-Time PCR Detection System (BioRad, CA, USA) were used for qPCR. Relative gene expression was normalized to *GAPDH* (Table 1).

**Table 1:** Primer sequences

Gene	Primer
*SOX9*	Forward: GGGCAAGCTCTGGAGACTTCTG
	Reverse: CTGCCCGTTCTTCACCGACT
*LDHB*	Forward: ACGCAGCTCACATTGTGTT
	Reverse: TGCCATTTTACACAGGAGGG
*MKI67*	Forward: ACTTTGGGTGCAACTTGACG
	Reverse: ACAACTCTTCCACTGGGACG
*WNT5A*	Forward: CTCGCCATGAAGCTGGAACT
	Reverse: GGTGCGACGAGAAGTGATCT
*LDHA*	Forward: TGACGTGCATTCCCGATTC
	Reverse: GCTGATCCTTCAGAGTTGCCA
*CTNNB1*	Forward: CAGTGCTAGGGTGCTGGAGTC
	Reverse: GCCATTGTCCACGCTGGATT
*GAPDH*	Forward: CATTTTCTCTTGCATCGCCAGGTG
	Reverse: GACTCCGACCTTCACCTTCCC

### Statistical analysis

GraphPad Prism 8 (GraphPad, San Diego) was used for statistical analysis. Paired or unpaired Student’s *t* test (parametric or nonparametric test) was used to analysis statistically significant treatment effects and defined statistical significance at *P* < 0.05 (*P < 0.05, **P < 0.01), and ns for no significance. Data was presented as mean ± SEM.

## Results

### SBM promotes hair regeneration and hair follicle development in cynomolgus monkeys

**Fig. 1:**
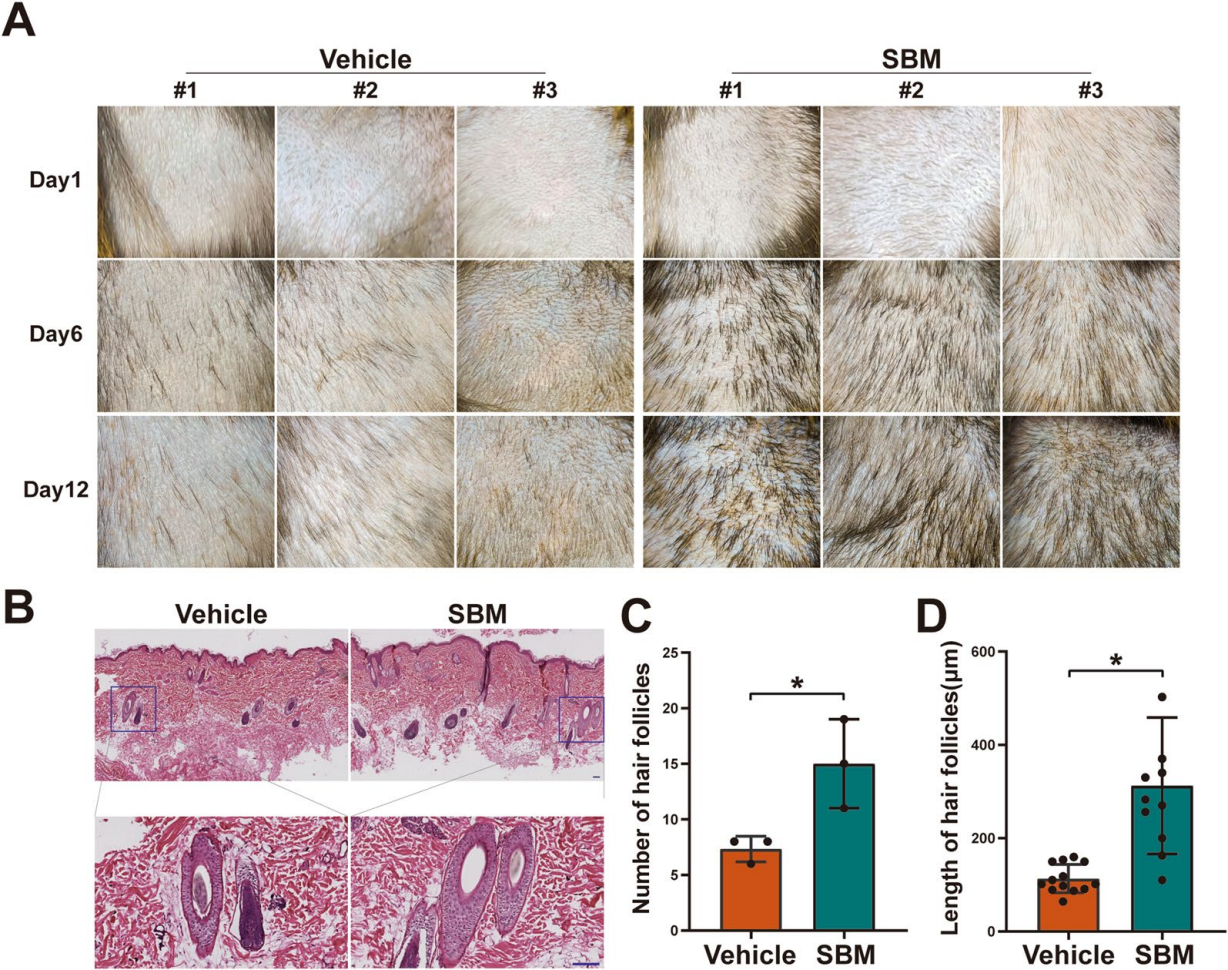
SBM promotes hair regeneration and hair follicle development in cynomolgus monkeys. **A** Changes of hair phenotypes over time in cynomolgus monkeys in different treatment groups (left: vehicle group, right: SBM group). (n = 6, 3 male cynomolgus monkeys and 3 female cynomolgus monkeys). **B** H&E stained of skin tissue obtained on day 12 from cynomolgus monkeys treated with vehicle or SBM daily. Scale bar: 100 μm. Quantitative statistics of the number (**C**) and longest length (**D**) of hair follicles in cynomolgus monkeys’ skin. (Magnification, 100 ×) *P < 0.05 vs. vehicle group

Initially, we administered SBM (SBM group) and vehicle solvent (vehicle group) on the back of cynomolgus monkeys and observed the hair growth phenotype. The results showed that hair regeneration in the shaved skin area was insignificant on the 6th and 12th day in the vehicle group, indicating that the 3-year-old cynomolgus monkeys were in the telogen phase of their hair-growth cycle. In contrast, in the SBM group, hair regeneration was significantly promoted (Fig. 1A). H&E staining analysis of the skin tissue showed that most hair follicles in the vehicle group were in a closed state, indicative of slow hair morphology development. However, the number of hair follicles in the SBM group increased significantly, with most follicles exhibiting a fusiform shape and connecting to the epidermis (Fig. 1B). These observations suggested that the hair follicles in the SBM group had entered the anagen phase, with a corresponding significant increase in the length and number of hair follicles (Fig. 1C, D). In conclusion, our findings suggest that SBM can promote hair regeneration and hair follicle development in cynomolgus monkeys.

### Cynomolgus monkey skin cell clusters are defined by scRNA-seq

**Fig. 2:**
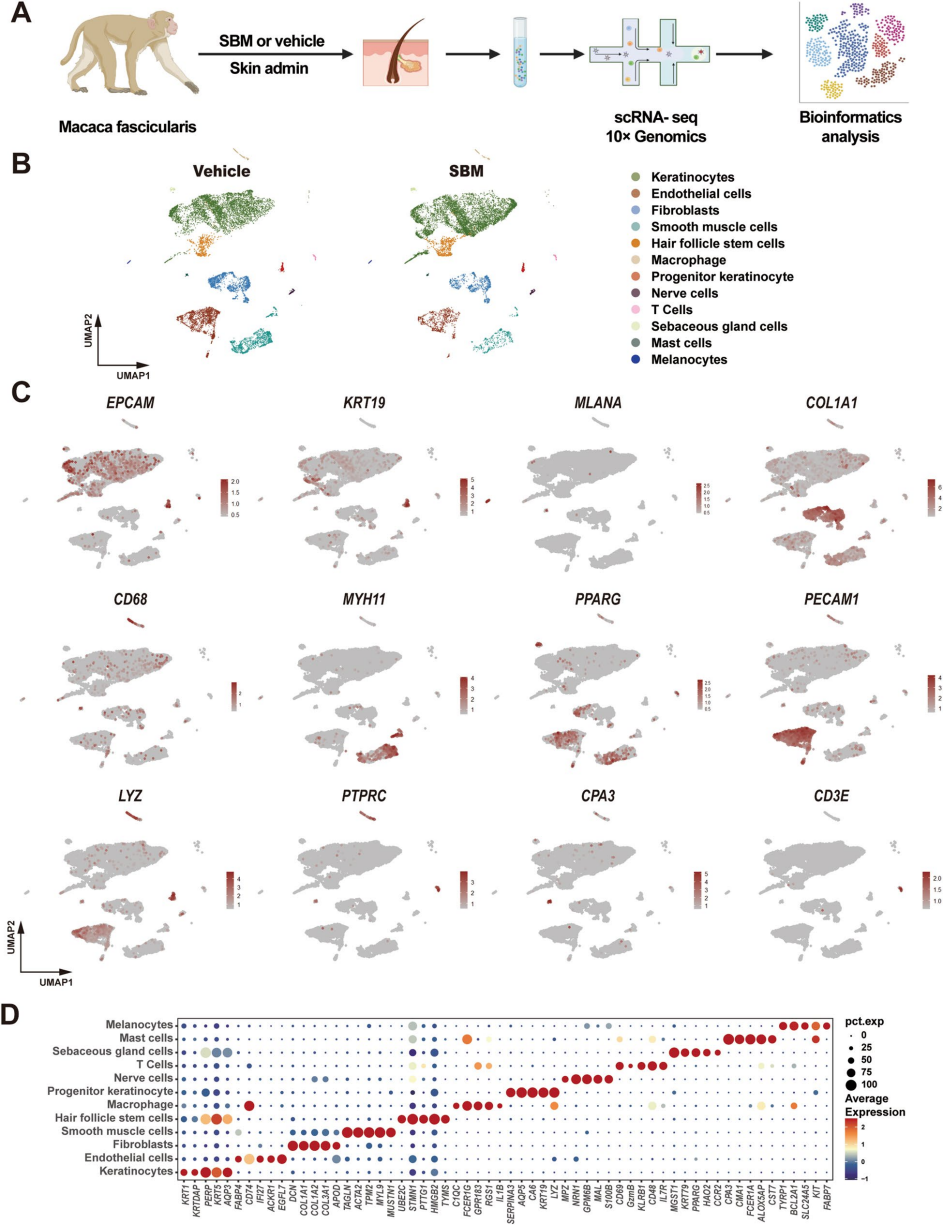
Cynomolgus monkey skin cell clusters defined by scRNA-seq. **A** Overview of the scRNA-seq experiment. SBM and vehicle solution were smeared twice daily for 12 days, and scRNA-seq was performed for the skin smeared of the same cynomolgus monkey with SBM and vehicle solution. **B** Uniform Manifold Approximation and Projection (UMAP) plot for dimension reduction of two paired samples cells colored by their cell type/identity. **C** Featureplot plots showing marker gene expression for cell type identification. Legend shows a color gradient of normalized expression. **D** Dotmap of top 5 differentially expressed genes (DEGs) across cell clusters. Red represents high expression; blue represents low expression

In order to further explore the mechanism how SBM promotes hair regeneration, skin tissue samples were collected from cynomolgus monkeys in both the vehicle group and the SBM group 12 days after smear administration. Droplet-based single cell isolation and 10 × Genomics single-cell RNA sequencing were performed (Fig. 2A). A total of 18,911 cells (including 10,001 in the vehicle group and 8910 in the SBM group) were obtained after filtering cells with nFeature number ranging from 500 to 6000 and mitochondrial genes proportion less than 5%. Clustering and UMAP dimensionality reduction were carried out, identifying 12 major cell clusters (Fig. 2B), including endothelial cells, fibroblasts, keratinocytes, macrophage, mast cells, melanocytes, HFSCs, nerve cells, progenitor keratinocytes, sebaceous gland cells, smooth muscle cells, and T cells. Notably, the major markers of cell cluster definition included *EPCAM* for identification of keratinocytes, *COL1A1* for fibroblasts, *PECAM1* for endothelial cells, *MYH11* for smooth muscle cells, *CPA3* for Mast cells, *PTPRC* for immune cells, *LYZ* for myeloid cells, *CD3E* for T cells, *MLANA* for melanocytes, and *PPARG* for sebaceous gland cells (Fig. 2C). The top 5 differential genes of cell clusters were visualized by dot plot (Fig. 2D).

### SBM up-regulates the expression of *SOX9* and *MKI67* in HFSCs

**Fig. 3:**
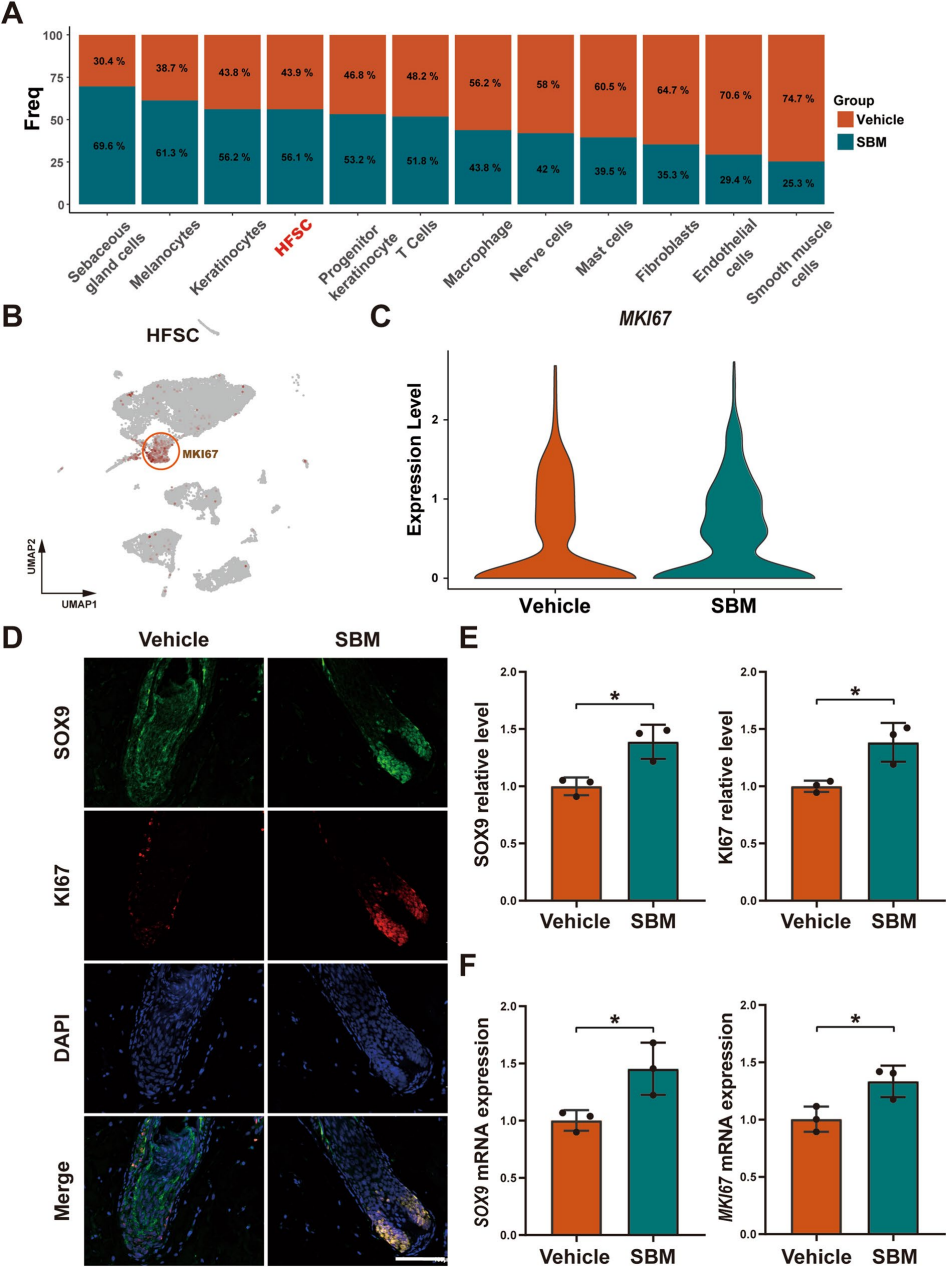
SBM up-regulates the expression of *SOX9* and *MKI67* in HFSCs. Bar charts of the proportion of cell clusters in the vehicle and the SBM group. **A** Feature plot of *MKI67* expression in total cells. **B** Violin plot depicting the expression of *MKI67* in the scRNA-seq of HFSCs with the vehicle and SBM group. **C** The expression of *MKI67* in violin plot for HFSCs. Immunofluorescence staining (**D**) and fluorescence quantitative analysis (**E**) of SOX9 and Ki67. Scale bar: 100 μm. (**F**) Relative mRNA expression level analysis of *SOX9* and *MKI67*. *GAPDH* was used for standardization. *P < 0.05 vs. vehicle group

Upon analysis of cell clusters ratios, the proportion of HFSCs was increased after SBM smear administration (Fig. 3A). MKI67, a proliferation-related marker gene [13] that functions closely with mitosis, was visualized and found to be specifically expressed in HFSCs (Fig. 3B). Furthermore, the expression of *MKI67* in HFSCs was significantly increased after SBM administration (Fig. 3C). SOX9, a nuclear transcription factor which produces hair follicle lineage cells [14], has been previously used as a marker of HFSCs [15], and could reflect the differentiation function. Through immunofluorescence staining in conjunction with fluorescence quantitative analysis, we found that the expression of SOX9 and KI67 in protein level was significantly increased in the SBM group (Fig. 3D, E), and the mRNA levels of *SOX9* and *MKI67* were also observed to be increased (Fig. 3F). In conclusion, our findings suggest that SBM has the capability to stimulate the activation of HFSCs and promote their ability to proliferate and differentiate.

### SBM regulates genes of HFSCs expressing differentially and extracellular martrix related pathway

**Fig. 4:**
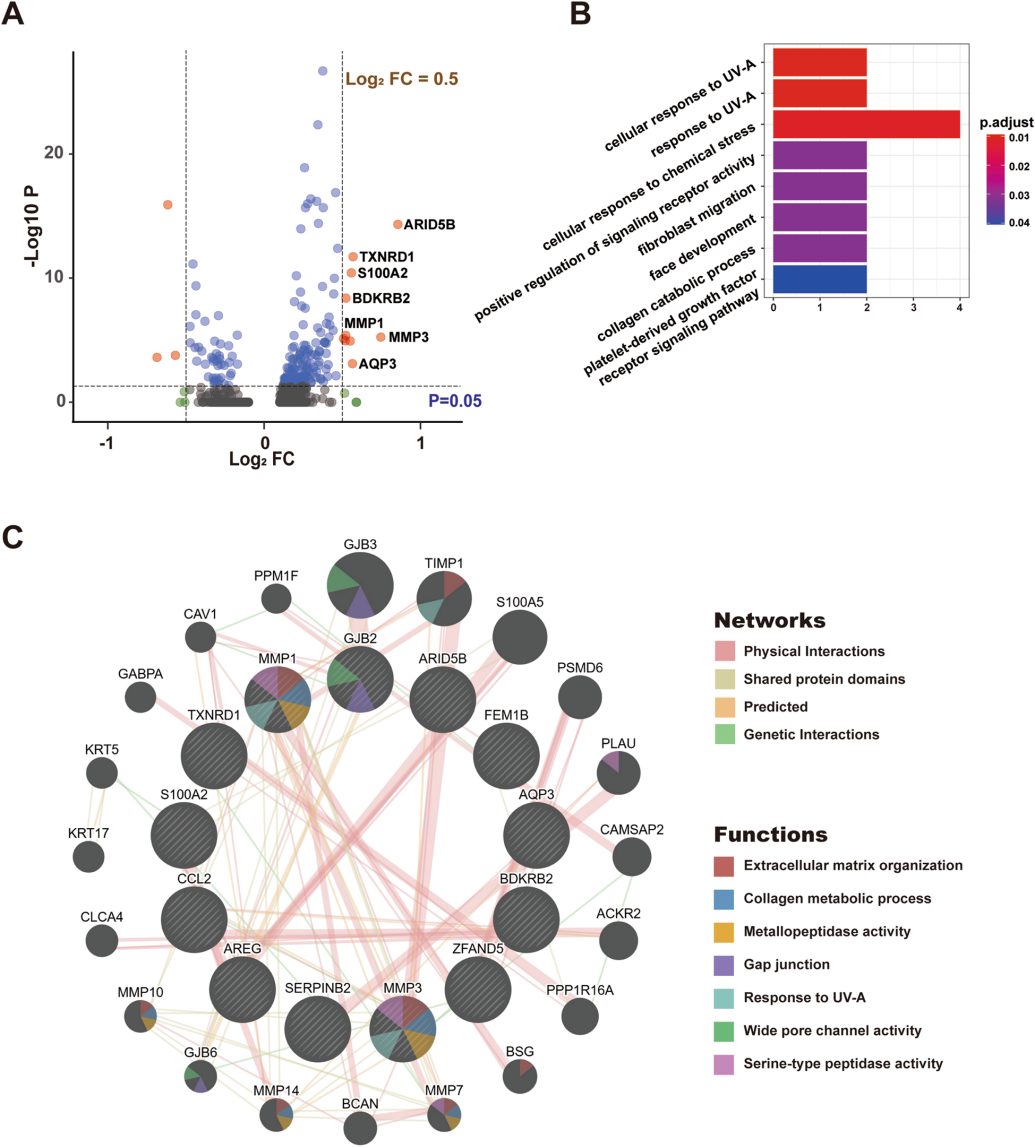
SBM regulates genes of HFSCs expressing differentially and extracellular martrix related pathway. **A** Volcano plot of differentially expressed genes in HFSCs between the vehicle and the SBM group. Significantly differentially expressed genes in the SBM group are shown as a red (up) or blue (down) dots. **B** Gene ontology analysis of differentially expressed genes. **C** Interaction network of differentially expressed genes

Differential gene expression analysis was conducted between the vehicle and the SBM group in HFSCs. The log_2_FC > 0.5 and the *p* value < 0.05 were set as the cut-off value to identify significantly up- and down-regulated genes. A total of 1562 differentially expressed genes were identified, with 13 up-regulated genes, including *ARID5B*, *MMP3*, *CCL2*, *SERPINB2*, *TXNRD1*, *AQP3*, etc., and 6 down-regulated genes including *TPPP3*, *MT1E*, *DDIT3*, *TAGLN*, *ACTA2*, *SCGB3A2*. These genes were visualized in a volcano plot (Fig. 4A). The Gene Ontology (GO) enrichment analysis of up-regulated genes showed that biological processes were “response to UV-A” and “collagen catabolic process” (Fig. 4B). The interaction network of differentially expressed genes revealed that the functions were mainly enriched in “extracellular martrix organization” and “collagen catabolic process” (Fig. 4C).

### SBM up-regulates the expression of *LDHA* in HFSCs

**Fig. 5:**
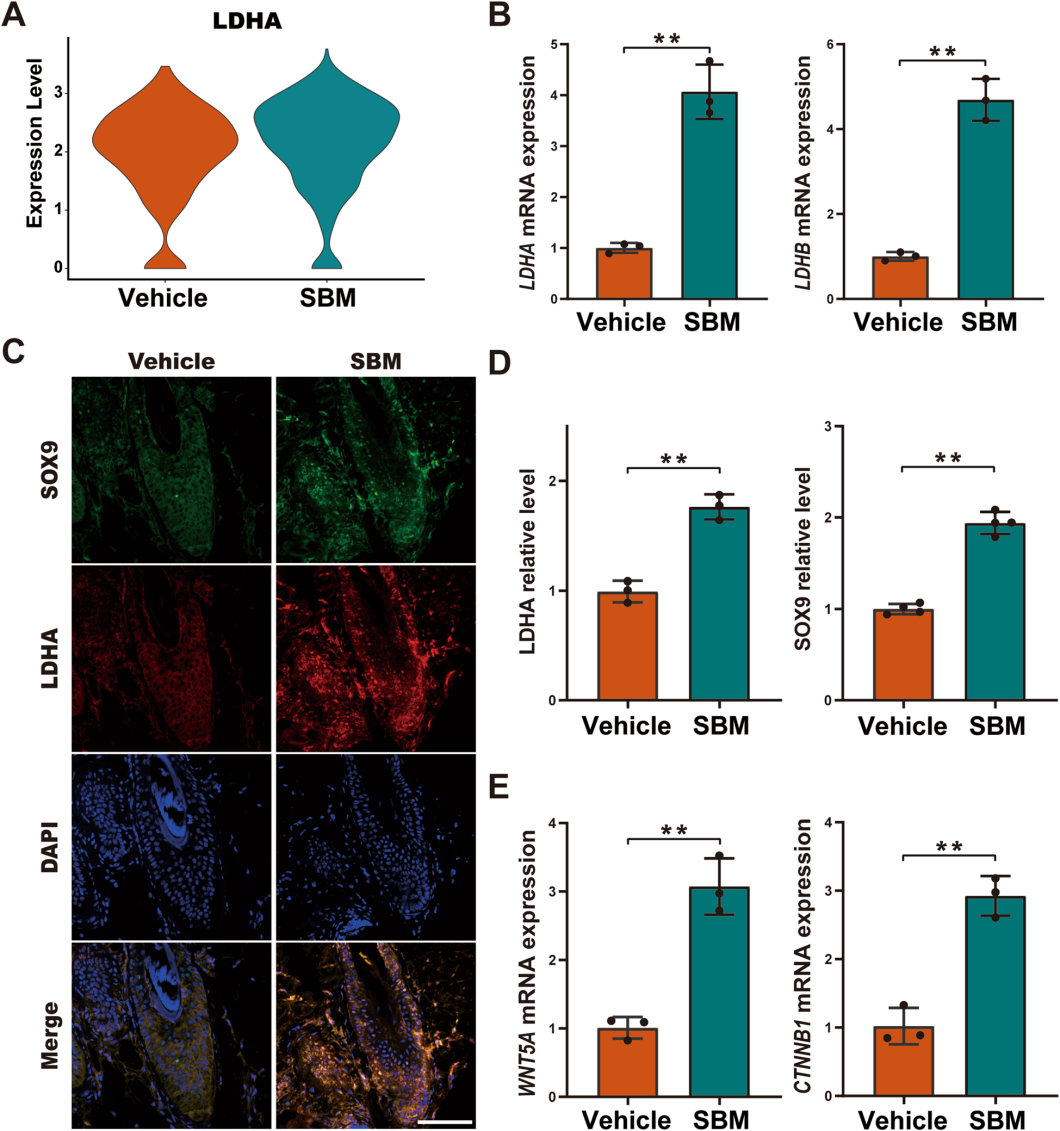
SBM up-regulates HFSCs lactic acid metabolism. **A** *LDHA* expression of HFSCs between the vehicle and the SBM group in violin plot. **B** Relative mRNA expression level analysis of *LDHA* and *LDHB*. *GAPDH* was used for standardization. Immunofluorescence staining (**C**) and fluorescence quantitative analysis (**D**) of SOX9 and LDHA. Scale bar: 100 μm. **E** Relative mRNA expression level analysis of *WNT5A* and *CTNNB1*. *GAPDH* was used for standardization. *P < 0.05, **P < 0.01 vs. vehicle group

Lactate dehydrogenase (Ldh) plays a crucial role in facilitating the reduction of pyruvate to lactate in glycolysis, which contributes to the production of essential metabolites and cell products necessary for proliferation. Previous studies have revealed that lactic acid metabolism is of great importance in HFSC activation [16, 17]. Furthermore, scRNA-seq data revealed that the expression of *LDHA* in HFSCs was significantly increased in the SBM group (Fig. 5A). Meanwhile, mRNA expression of both *LDHA* and *LDHB* was also significantly increased in the SBM group (Fig. 5B). Additionally, through immunofluorescence staining combined with fluorescence quantitative analysis, we found that SBM could improve the LDHA protein expression in HFSCs (Fig. 5C, D). Notably, SBM also increased the mRNA expressions of *WNT5A* and *CTNNB1* in skin tissue, indicating its potential regulatory role in the WNT pathway to promote the activation and proliferation of HFSCs (Fig. 5E). To conclude, SBM may facilitate the activation of HFSCs by regulating lactate metabolism.

### HFSCs with high expression of *LDHA* regulates autophagy-related pathways

**Fig. 6:**
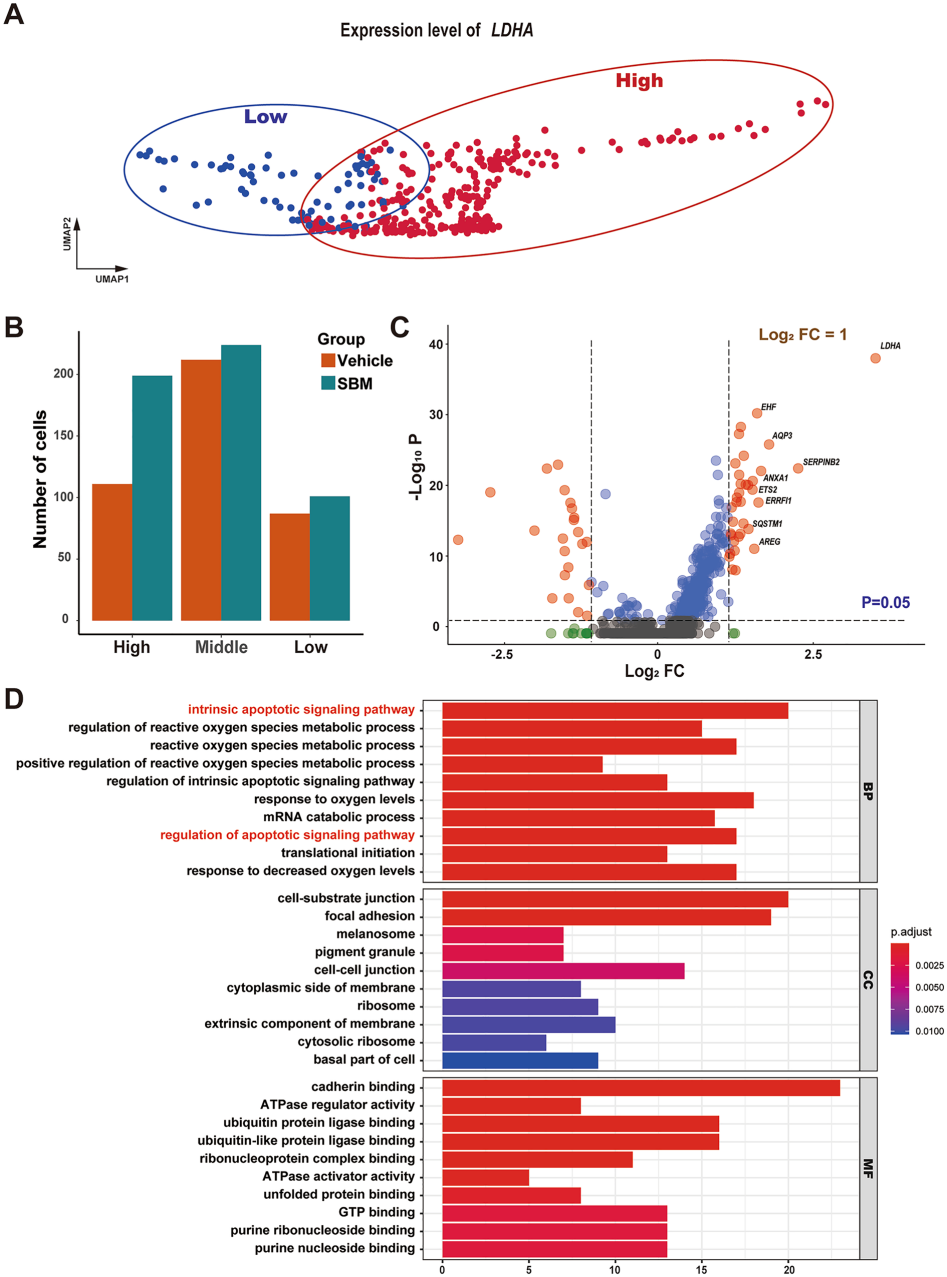
HFSCs with high expression of *LDHA* regulates autophagy related pathways. **A** The distribution of HFSCs with low and high *LDHA* expression in UMAP plot. HFSCs with *LDHA* expression > 2.5 (High) and *LDHA* expression < 1 (Low). **B** The proportion of HFSCs with high, middle and low *LDHA* expression. **C** Volcano plot of differentially expressed genes in HFSCs between low and high expression of *LDHA*. Significantly differentially expressed genes in the SBM group are shown as a red (up) or blue (down) dots. **D** Gene ontology analysis of differentially expressed genes

To investigate the mechanism of LDHA affecting HFSC activation, HFSCs were divided into three groups according to *LDHA* expression level: *LDHA*^*High*^ HFSCs (*LDHA* expression > 2.5), *LDHA*^*Mid*^ HFSCs (1 ≤ *LDHA* expression ≤ 2.5) and *LDHA*^*Low*^ HFSCs (*LDHA* expression < 1). *LDHA*^*High*^ HFSCs and *LDHA*^*Low*^ HFSCs could be clearly distinguished (Fig. 6A). *LDHA*^*High*^ HFSCs were primarily affected by SBM, its number significantly increased in the SBM group (Fig. 6B). Further, differential gene expression analysis of scRNA-seq data was performed for HFSCs between the vehicle and the SBM groups. Significantly up- and down-regulated genes were identified with cut-off values of log_2_FC > 1 and *p* value < 0.05, resulting in a total of 1399 differentially expressed genes, among which 38 genes were up-regulated, including *SERPINB2*, *AQP3*, *ZFAND5*, *ERRFI1*, *EHF*, etc., and 32 genes were down-regulated including *PDXP*, *COX1*, *COL1A1*, *RAMP1*, *CYTB*, etc. These genes were visualized via the volcano plot (Fig. 6C). GO enrichment analysis of the up-regulated genes showed that biological processes (BP) were mainly in autophagy-related signaling pathways and reactive oxygen species metabolic process. While “reactive oxygen species metabolic process” is correlated to *LDHA* based differentially expressed genes, and autophagy may participate in HFSC activation. Cell components (CC) were primarily associated with cell-substrate junctions and cell–cell junctions (Fig. 6D).

### Cell interaction of laminin pathway is enhanced in the SBM group

**Fig. 7:**
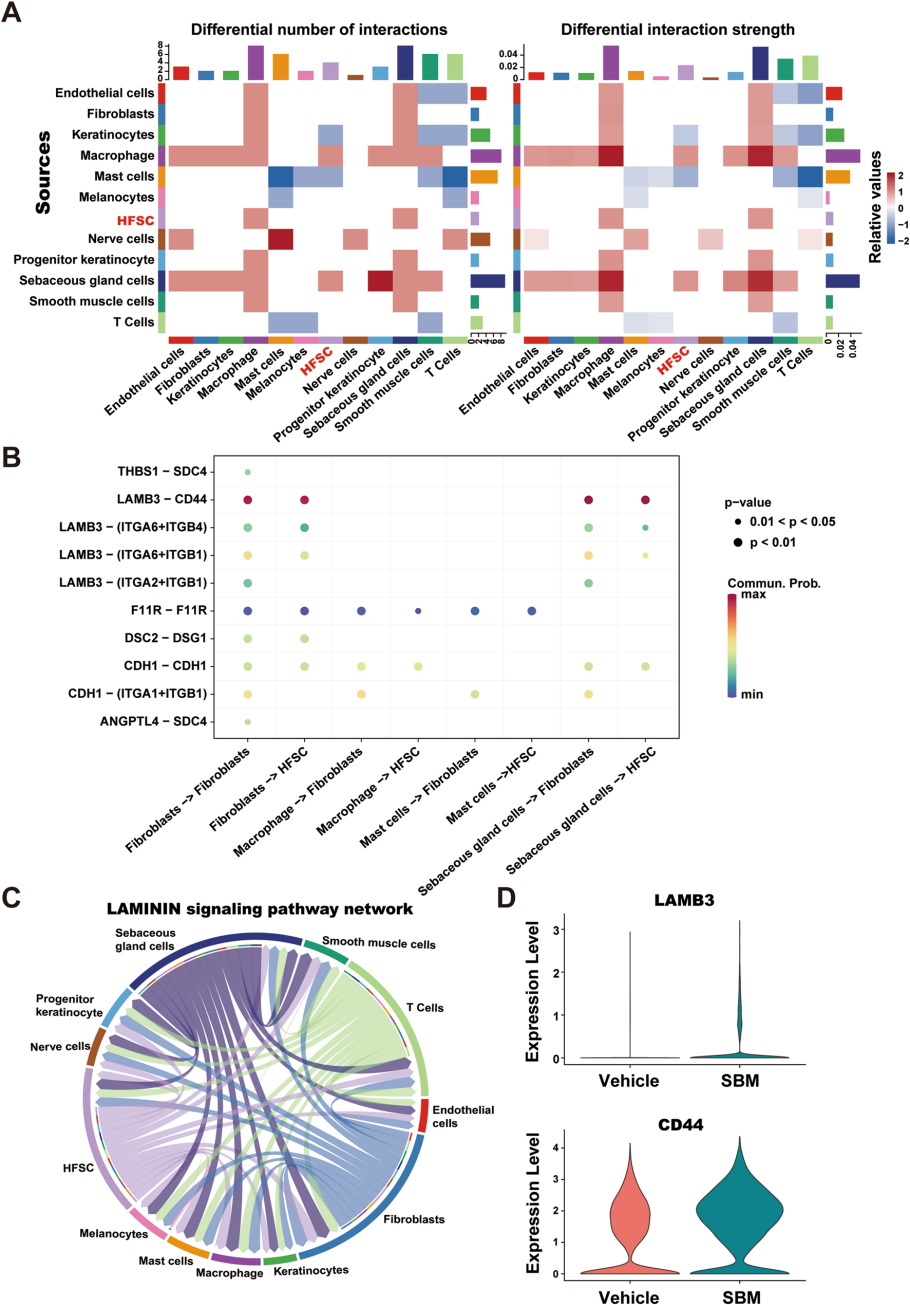
Cell interaction of laminin pathway is enhanced in the SBM group. **A** Heatmap of differential number of interactions and differential interaction strength. Red refers higher level in the SBM group and blue refers lower in the SBM group. **B** Dot plot of differential ligand-receptor pairs. **C** Intercellular communication networks of the Laminin signaling pathway. The color of each line is consistent with the signal sender. The circumference of the circle refers to the strength of the interaction with other cells. **D** *LAMB3* and *CD44* expression in total cells between the vehicle and the SBM group in violin plot

To gain a deeper understanding of the mechanism underlying HFSC activation induced by SBM, we conducted cell interaction analysis of ligand-receptor pairs [18]. Our results demonstrated that the interaction between HFSCs and various cell types was enhanced in the SBM group (Fig. 7A). More specifically, the laminin pathway interaction, represented by CD44-LAMB3, was enhanced between HFSCs and keratinocytes, as well as between HFSCs and fibroblasts (Fig. 7B, C). The scRNA-seq data revealed a significant increase in the expression of *LAMB3* and *CD44* in the SBM group (Fig. 7D). It appears that SBM may be involved in HFSC activation by affecting the laminin pathway in intercellular adhesion.

**Fig. 8:**
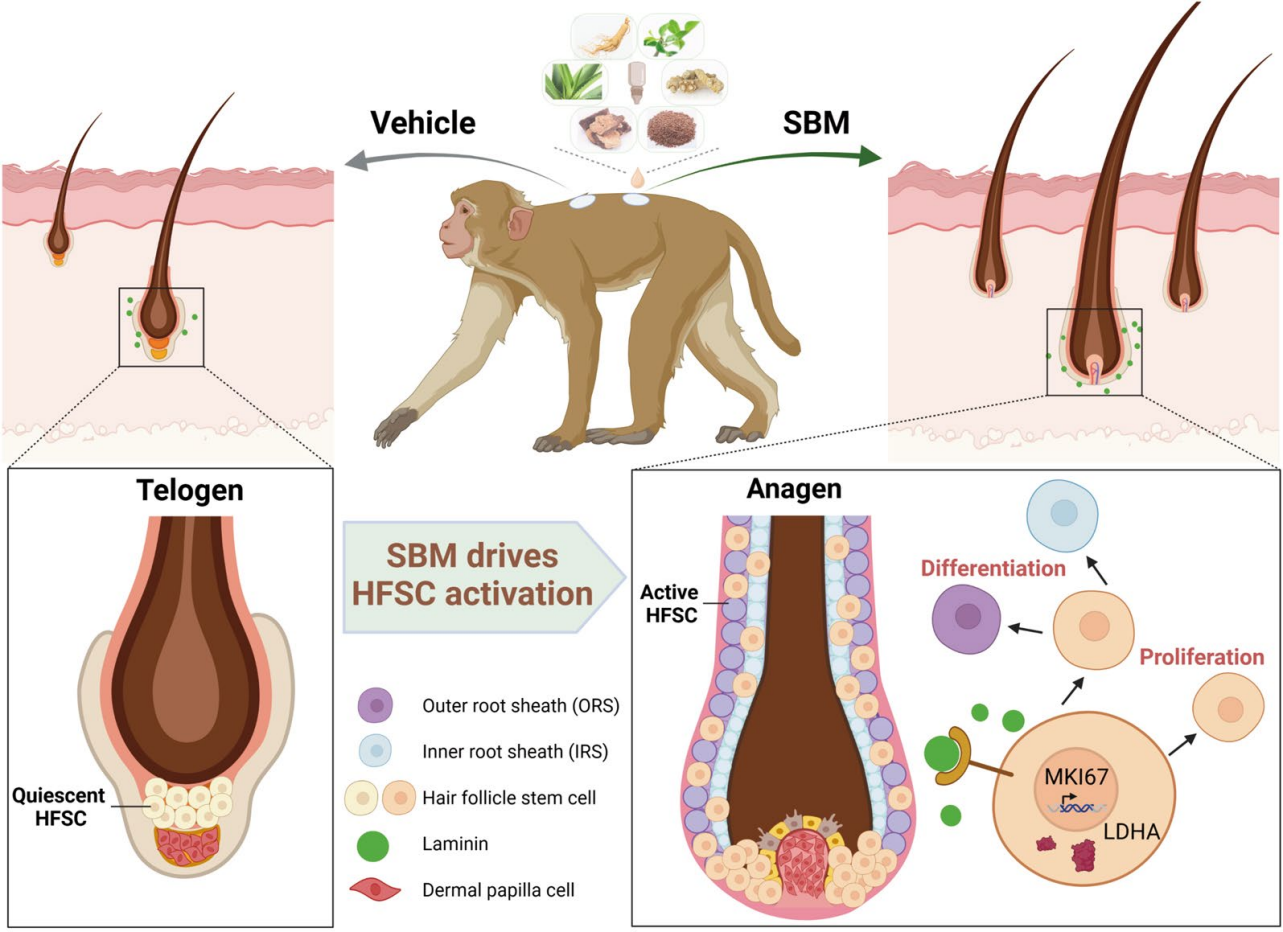
Traditional Chinese medicine SBM regulates lactic acid metabolism and drives hair follicle stem cell activation to promote hair regeneration. SBM consists of a variety of traditional Chinese medicine, which could shift hair growth cycle from the telogen phase to the anagen phase and promote hair regeneration. Mechanically, SBM could up-regulate *LDHA* expression and drive HFSC activation for proliferation and differentiation

In conclusion, we confirm that SBM can promote hair regeneration in cynomolgus monkeys. Mechanically, SBM can up-regulate LDHA expression and drive HFSC activation for proliferation and differentiation (Fig. 8).

## Discussion

The activation of HFSCs and the development of hair follicles are regulated by various hormones [19], growth factors (GFs) [20], and bone morphogenetic protein (BMP). In addition, many transcription factors (TFs), such as SOX9, play a critical role in the rate of gene transcription and the regulation of various signaling pathways, including Wnt/β-Catenin, Notch, Sonic Hedgehog (Shh) and BMP, to maintain HFSCs stemness. The absence of SOX9 during embryonic skin development results in the inability to form HFSCs [21, 22]. Although SOX9 is not directly involved in the activation of HFSCs, the absence of SOX9 causes HFSCs to lose their stemness and differentiate along the epidermal lineage, and the mechanism is mainly related to activin signal [15]. The Wnt/β-catenin signaling pathway is vital for hair regeneration [23, 24], and the up-regulation of this pathway can promote HFSCs transitioning from quiescent to an active state. The Shh pathway promotes the proliferation of quiescent HFSCs, which is closely related to hair morphogenesis [25]. Meanwhile, the BMP pathway antagonizes Wnt signaling and inhibits HFSC proliferation, which maintains HFSCs in the quiescence state [26]. The intricate network formed through multiple pathways determines the cell-fate of HFSCs during hair cycle [27–29].

Metabolism plays a crucial role in activating HFSCs [14]. Glucose is extracted from the blood and metabolized it into pyruvate in HFSCs. Subsequently, pyruvate is either transported to the mitochondria to produce energy, or it is utilized by lactate dehydrogenase to generate lactic acid. The glycolysis pathway plays a critical role in cell proliferation by providing the necessary substrates and energy for the process. Previous researches have revealed that lactate generation is essential for the activation of HFSCs, and deletion of *LDHA* can prevent their activation [17]. UK-5099, known as a mitochondrial pyruvate carrier 1 (MPC1) inhibitor, inhibits the mitochondrial pyruvate carrier pharmacologically and promotes lactate production, resulting in a robust acceleration of the hair cycle [30].

Autophagy is intricately related to hair follicle development. The level of autophagy is enhanced in the anagen phase and maintains a low level in the telogen phase [31, 32]. Metformin activates the adenosine monophosphate (AMP)-activated protein kinase (AMPK) to reduce energy intake and inhibit the respiratory chain, thereby inducing autophagy, shifting the hair cycle from the telogen phase to the anagen phase.

Laminins are large groups of extracellular α, β, and γ chain trimers in the basement membrane (BM) of skin. During hair follicle development, the composition of laminins in the basement membrane zone (BMZ) changes [33]. LN-332 is abundant beneath the interfollicular epidermis, whereas LN-511 is most abundant at the lower part of the hair follicle. The various components of laminin have a complex effect on HFSCs. Previous studies have revealed that LN-332 suppresses Wnt signaling, whereas LN-511 promotes TGF-β signaling. The overexpression of LN-511, however, can cause aberrant HFSC activation, leading to the depletion of HFSCs. The precise ratio of LN-511 and LN-332 regulates the core HFSCs fate-determining signaling pathways, highlighting the importance of extracellular matrix (ECM) niche in HFSC activation [34].

The primary constituents of SBM include epigallocatechin gallate (EGCG), epicatechin gallate (ECG), stilbene glycoside (TSG), and various ginsenoside (ginsenoside Rg1, ginsenoside Rf, ginsenoside Rc and ginsenoside Rb2, ginsenoside Rd and ginsenoside Rb1). Previous researches reveal that EGCG ameliorates androgenic alopecia by selectively inhibiting 5α-reductase activity, and up-regulating phosphorylated Erk and Akt and increasing the Bcl-2/Bax ratio on DPCs [35]. Various ginsenosides in ginseng have been reported to regulate key targets in hair growth, including DKK1, SHH, VEGF, TGF-β, MMPs, etc. [36]. Ginsenoside Rg3 can up-regulate the expression of vascular endothelial growth factor (VEGF) in DP cells, and drive HFSC activation [37]. Polygonum multiflorum extract can also promote hair growth by activating DP cells to prolong the anagen period and delay the telogen period. Various components in SBM coordinate and interact with each other, acting on AGA and multiple links in hair regeneration.

As Non-human primates (NHP), cynomolgus monkeys exhibit a range of human-like characteristics, not only genetically, but also in organ development, physiological function, pathological response and biochemical metabolism [38, 39], thus are valuable experimental animal models for medical research and drug development. In particular, they play a pivotal role in preclinical pharmacological and toxicological research for COVID-19 [40–42]. Our research in cynomolgus monkeys reveals that SBM can drive HFSC activation by up-regulating LDHA-mediated lactic acid metabolism, thereby promoting hair regeneration. The mechanism may be associated with autophagy regulation and Laminin pathway-mediated cell adhesion.

## Conclusion

Our research unveils that SBM can drive HFSC activation by up-regulating LDHA-mediated lactic acid metabolism, which in turn promotes hair regeneration in cynomolgus monkeys. This study provides a scientific explanation for the promotion of hair regeneration by traditional Chinese Medicine SBM.

## Data Availability

The single-cell transcriptomic data is uploading to the GEO database. And other original data presented in the study are included in the article, further inquiries can be directed to the corresponding author.

## Abbreviations

SBM: Shi-Bi-Man
scRNA-seq: Single-cell RNA sequencing
qPCR: Quantitative polymerase chain reaction
HFSC: Hair follicle stem cell
AGA: Androgen alopecia
FDA: United States Food and Drug Administration
EGCG: Epigallocatechin gallate
TSG: Stilbene glycoside
PBS: Phosphate-buffered saline
PCA: Principal component analysis
UMAP: Uniform manifold approximation and projection
DEGs: Differentially expressed genes
H&E: Hematoxylin and eosin
BSA: Bovine serum albumin
HRP: Horseradish peroxidase
GO: Gene Ontology
LDH: Lactate dehydrogenase
BP: Biological process
CC: Cell component
GFs: Growth factors
BMP: Bone morphogenetic protein
TFs: Transcription factors
Shh: Sonic Hedgehog
AMP: Adenosine monophosphate
BMZ: Basement membrane zone
ECM: Extracellular matrix
NHP: Non-human primate
