# Correction: Traditional Chinese Medicine Shi-Bi-Man regulates lactic acid metabolism and drives hair follicle stem cell activation to promote hair regeneration

**DOI:** 10.1186/s13020-023-00807-8

**Published:** 2023-08-01

**Authors:** Haojie Du, Tao Zhang, Qiao Wang, Xinran Cao, Huiwen Zheng, Jiabin Li, Jianxia Zhu, Jiao Qu, Lehang Guo, Yang Sun

**Affiliations:** 1 State Key Laboratory of Pharmaceutical Biotechnology, School of Life Sciences, Nanjing University, 163 Xianlin Avenue, Nanjing, 210023 China; 2 Department of Ultrasound, Shanghai Tenth People’s Hospital, Shanghai, China; 3 Department of Dermatology, Children’s Hospital, Zhejiang University School of Medicine, National Clinical Research Center for Child Health, Hangzhou, 310052 Zhejiang China; 4 Shenzhen Sipimo Technology Co., Ltd., Shenzhen, 518000 Guangdong China; 5 Jiangsu Key Laboratory of New Drug Research and Clinical Pharmacy, Xuzhou Medical University, 209 Tongshan Road, Xuzhou, 221004 China

**Correction: Chinese Medicine (2023) 18:84** 10.1186/s13020-023-00791-z

Following publication of the original article [1], the authors reported an error in Fig. 6D. The correct Fig. 6 has been provided in this Correction.

**Fig. 6:**
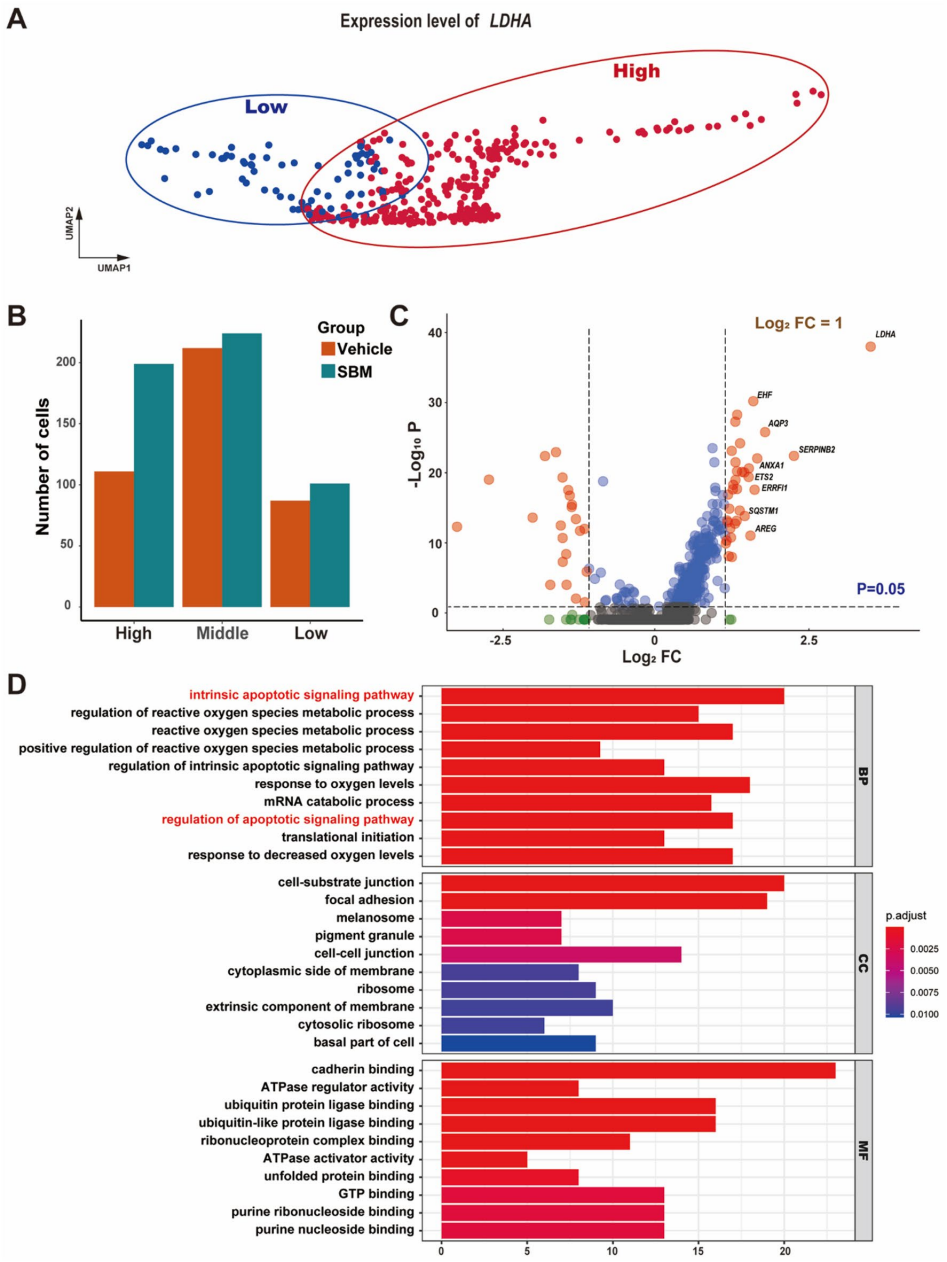
HFSCs with high expression of *LDHA* regulates autophagy related pathways. **A** The distribution of HFSCs with low and high *LDHA* expression in UMAP plot. HFSCs with *LDHA* expression > 2.5 (High) and *LDHA* expression < 1 (Low). **B** The proportion of HFSCs with high, middle and low *LDHA* expression. **C** Volcano plot of differentially expressed genes in HFSCs between low and high expression of *LDHA*. Significantly differentially expressed genes in the SBM group are shown as a red (up) or blue (down) dots. **D** Gene ontology analysis of differentially expressed genes

The original article [1] has been corrected.
